# Molecular Chromophore-DNA Architectures With Fullerenes: Optical Properties and Solar Cells

**DOI:** 10.3389/fchem.2021.645006

**Published:** 2021-02-23

**Authors:** Sara Müller, Felix Manger, Lorenz Graf von Reventlow, Alexander Colsmann, Hans-Achim Wagenknecht

**Affiliations:** ^1^ Institute of Organic Chemistry, Karlsruhe Institute of Technology, Karlsruhe, Germany; ^2^ Material Research Center for Energy Systems, Karlsruhe Institute of Technology, Karlsruhe, Germany; ^3^ Light Technology Institute, Karlsruhe Institute of Technology, Karlsruhe, Germany

**Keywords:** pyrene, nile red, fluorescence, solid-state circular dichroism, oligonucleotide

## Abstract

Supramolecular chemistry allows the construction of complex molecular architectures and the design of collective photophysical properties. DNA is an attractive template to build such supramolecular architectures due to its helical structure, the defined distances between the bases and the canonical base pairing that results in precise control of the chromophore position. The tailored properties of DNA-templated supramolecules eventually allow their implementation into optoelectronic applications. For the generation of free charge carriers from photo-generated excitons, fullerenes can be utilized. We synthesized two fullerene derivates, one of which binds by electrostatic interactions to single-stranded DNA, while the other contains two 2′-deoxyuridine moieties and assembles specifically along oligo-2′-deoxyadenosines (dA_20_) as DNA template. The DNA-directed assembly of both fullerenes in aqueous solution was investigated by UV/Vis absorbance and circular dichroism (CD) spectroscopy. The specific interactions with DNA make fullerenes with the 2′-deoxyuridine moieties a significantly better component for supramolecular DNA architectures. We studied the fluorescence quenching of both fullerenes with a DNA chromophore assembly. To investigate one of the key properties for optoelectronic applications, that is the supramolecular structure of the DNA-based assemblies in the solid phase, we characterized the CD of supramolecular chromophore-DNA architectures in thin films. Remarkably, the helical chirality of the chromophore assemblies that is induced by the DNA template is conserved even in the solid state. Upon implementation into organic solar cells, the external quantum efficiency measurements showed charge carrier generation on all three chromophore components of the DNA assemblies. The fullerenes with the 2′-deoxyuridine moieties enhance the quantum efficiency of the conversion process significantly, demonstrating the potential of DNA as structural element for ordering chromophores into functional π-systems, which may be employed in future organic solar cells.

## Introduction

Supramolecular chemistry enables the construction of complex molecular architectures and functional hybrids (Amabilino et al., 2017). Collective photophysical properties are achieved especially by supramolecular multi-chromophore architectures, which can be used, e.g. for light harvesting (Stewart and Fox, 1996), in solar cells (Günes et al., 2007) or as organic semiconductors (Günes et al., 2007). For such purposes, a clearly defined, hierarchically ordered arrangement of chromophore assemblies can be beneficial in order to tailor the optoelectronic properties. In this context, DNA is an attractive template to build such supramolecular architectures. In particular, the helical structure as well as the defined distances between the bases and the canonical base pairing precisely control the position of the chromophores and thus suppresses self-quenching that is typically obtained in uncontrolled chromophore aggregates (Ensslen and Wagenknecht, 2015; Vybornyi et al., 2019). Modified nucleosides and 2′-deoxynucleosides assemble porphyrins (Sargsyan et al., 2013; Sargsyan et al., 2014; Ishutkina et al., 2018), naphthalenes (Janssen et al., 2009a; Janssen et al., 2009b; Stevens et al., 2011) and pyrenes (Sezi and Wagenknecht, 2013) successfully along single-stranded DNA templates. We realized bichromophore-DNA architectures in a non-covalent and sequence-selective way. Pyrenes and nile reds (Ensslen et al., 2015; Hofsäß et al., 2018) or pyrenes and perylenes (Müller et al., 2020) work both as energy transfer pairs. By attaching two dyes to two different nucleosides the optical properties could be programmed by the sequence of the DNA template. So far, such DNA chromophore architectures were only characterized by optical spectroscopy in solution.

**FIGURE 1:**
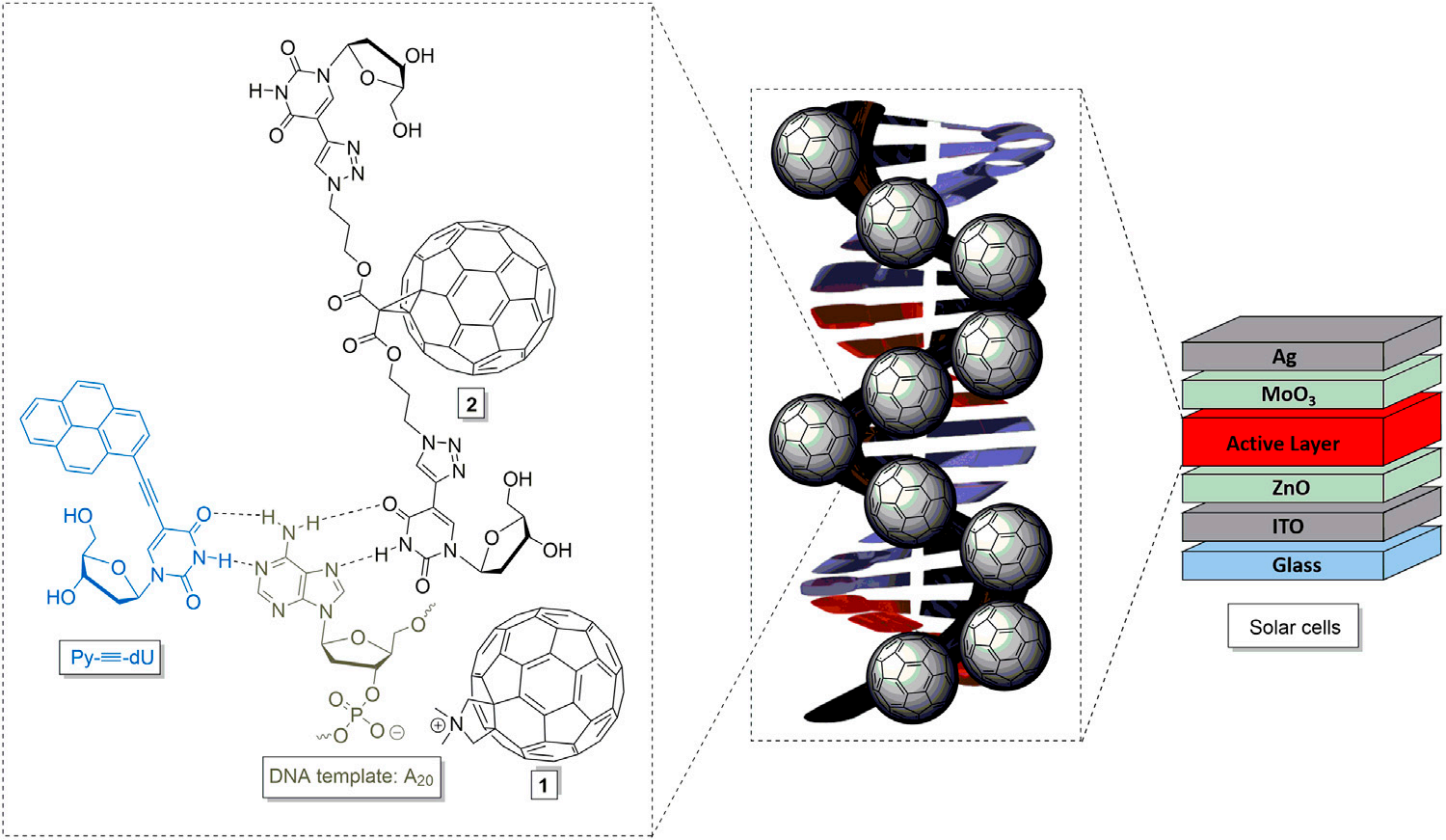
Modified fullerenes **1** and **2**, and their proposed interactions with DNA-templated chromophore assemblies, representatively drawn for Py-≡-dU and oligo-2′-deoxyadenosines (dA_20_), to yield DNA-based molecular architectures and materials for solar cells.

For the integration of these chromophores into organic optoelectronic applications such as solar cells, charge carrier separation and transport play an important role. Organic solar cells feature an interpenetrating network of two semiconductors, i.e. an electron donor and an electron acceptor (bulk-heterojunction, BHJ) for light harvesting, the latter of which is often realized using fullerene derivatives (Sariciftci et al., 1992; Liu et al., 2014; Zhao et al., 2016; Rasool et al., 2019). Although recent high performance organic solar cells with power conversion efficiencies of up to 17% employed non-fullerene acceptors (Cui et al., 2020; Guo et al., 2020; Liu et al., 2020), fullerene derivatives are still regularly used, e.g., on industrial scale or as a third component in ternary semiconductor mixtures(Pan et al., 2019; Chen et al., 2021). The supramolecular order of organic solar cells is critical for charge carrier separation and transport (Gurney et al., 2019; Zhao et al., 2020). Therefore, several attempts have been made to gain control of the supramolecular order of polymer-fullerene solar cells either by attaching the fullerene covalently or by intermolecular interactions (Troshin et al., 2007; Miyanishi et al., 2010; Sary et al., 2010; Lin et al., 2012; Tevis et al., 2012). Here, the templating function of DNA can provide a handle toward tailored molecular order. In order to use supramolecular DNA architectures as light-harvesting layers in solar cells, fullerenes must be conjugated to nucleosides or oligonucleotides. However, covalent fullerene-conjugates with nucleosides and DNA are only rarely found in literature. One of the earliest example was published in 1994 by Hélène et al. for sequence-selective photoinduced DNA cleavage (Boutorine et al., 1995). A few other nucleoside- or oligonucleotide-fullerene adducts followed for biological and photobiological applications (Boutorine et al., 1995; An et al., 1996; Bergamin et al., 2001; Ros et al., 2002; Yang et al., 2008). A covalent fullerene-guanosine adduct assembles a fullerene-porphyrine dyad by Watson-Crick base pairing (Sessler et al., 2005). The covalent conjugation of fullerene to the DNA template yielded a DNA-based light-harvesting system through the non-covalent assembly of pyrene and nile red dyes, which was used for the first time as an active layer in an organic solar cell (Ensslen et al., 2016). This showed that supramolecular DNA chromophore architectures are suitable for the use in optoelectronic applications. Herein, we report a new 2′-deoxyuridine conjugate fullerene **2** which we compare with a literature-known fullerene **1** (Cassell et al., 1998) that both assemble along DNA templates (Figure 1) by two different types of interactions, as well as the spectroscopic investigation of the resulting DNA-based light harvesting systems with pyrene- and nile red-nucleoside conjugates Py-≡-dU and Nr-≡-dU. Using circular dichroism measurements, we investigated whether the DNA-templated chromophore assembly prevailed the transition to the solid state upon thin film drying, eventually enabling their implementation as light-harvesting compounds in organic solar cells.

## Materials and Methods

All materials, methods and experimental procedures are described in the Supplementary Information.

## Results and Discussion

### Synthesis of a New Fullerene-2′-deoxyuridine Conjugate and Its Optical Properties

**FIGURE 2:**
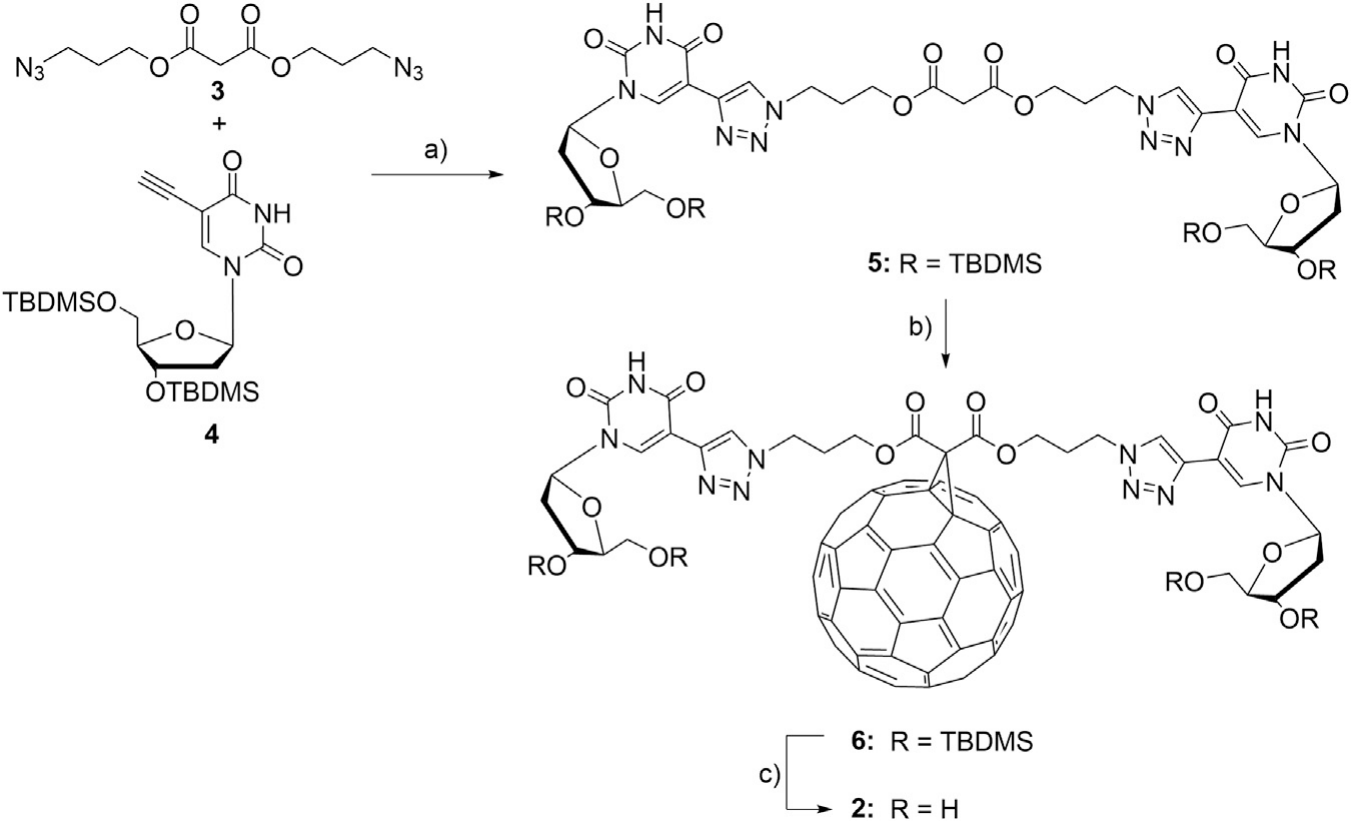
Synthesis of fullerene-2′-deoxyuridine conjugate **2**: a) sodium ascorbate, TBTA, Cu(MeCN)_4_PF_6_, CH_2_Cl_2_/DMF, r.t., 16 h, 92%; b) C_60**,**_ DBU, I_2_, toluene, r.t., 16 h, 26%; c) Et_3_N·3HF, DMF, r.t., 16 h, 75%.

In order to equip fullerenes with motifs for binding to DNA components, we synthesized the two modified fullerenes **1** and **2** (Figure 2). Fullerene **1** contains a positive charge at the methylated pyrrolidine moiety and therefore binds to DNA by pure electrostatic interactions. Fullerene **2** is conjugated to two 2′-deoxyuridines that are able to bind to complementary 2′-deoxyadenosines by canonical base pairing mediated by hydrogen bonding. Fullerene **1** was published by Tour et al. and was prepared according to those published procedures (Cassell et al., 1998). For fullerene **2**, we chose the broadly applied malonic ester to conjugate the 2′-deoxyuridines to the fullerene (Freitas et al., 2008). The malonic ester **3** is equipped with one azide groups at each end (Abellán Flos et al., 2016). The copper(I)-catalyzed cycloaddition between **3** and 5-ethynyl-2′-deoxyuridine **4** (Hornum et al., 2015) followed our published procedure for DNA modification (Berndl et al., 2009) but in a different solvent mixture (CH_2_Cl_2_/DMF) and gave the doubly nucleoside-modified malonic ester **5** in 92% yield. **5** was subsequently conjugated to C_60_-fullerene in 26% yield. Finally, the TBDMS protecting groups at the 3′- and 5′-positions of adduct 6 were removed to obtain the fullerene-(dU)_2_ conjugate 2 in 75% yield.

**FIGURE 3:**
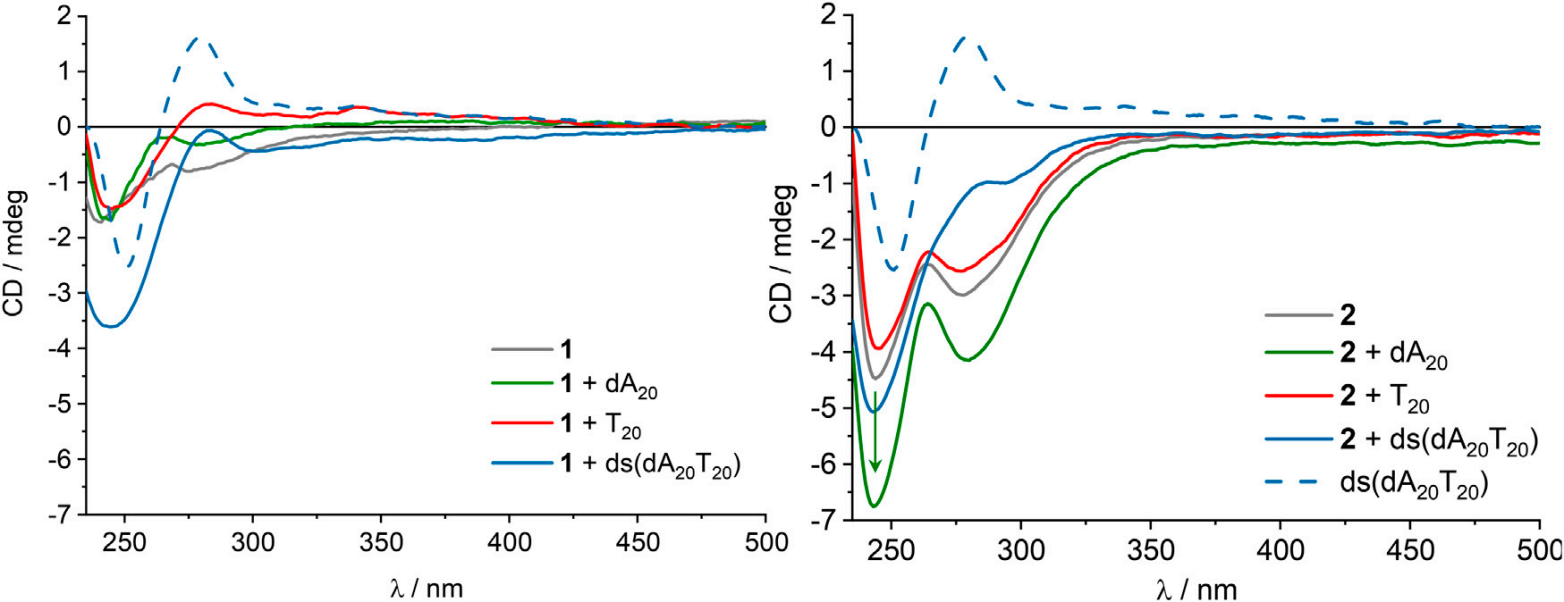
CD of **1** **(left)** and **2** **(right)**, each 15.0 µM in aqueous solution (2% DMSO), and in the presence of 0.75 μM dA_20_, 0.75 μM T_20_ or 0.75 μM ds (dA_20_T_20_), and reference spectrum of 0.75 μM ds (dA_20_T_20_).

The assembly of the fullerenes **1** and **2** in the presence of single-stranded DNA templates in aqueous solution was followed by UV/Vis absorption (see Supplementary Figure S1) and CD spectroscopy (Figure 3 and Supplementary Figure S2). Fullerenes **1** and **2** were separately dissolved as 2.00 mM stock solutions in DMSO, and were added to 0.75 μM dA_20_ as DNA template complementary to the 2′-deoxyuridine in water. Control experiments were performed with 0.75 μM T_20_ as nonspecific DNA template that does not provide complementary binding sites for fullerene **2**. Finally, we obtain 15.0 μM concentration of **1** or **2**, respectively, and 2% DMSO as cosolvent in the aqueous solution which is typically tolerated by the canonical base pairing between nucleosides and base pair stacking in DNA. The CD of fullerene **1** is weak and is not intensified by the presence of the single-stranded templates dA_20_ and T_20_. Hence, fullerene **1** neither forms a chiral assembly itself in aqueous solution nor with the single-stranded templates dA_20_ and T_20_ because only electrostatic interactions prevail. In contrast to double-stranded DNA, single stranded DNA typically has no helical order. With double-stranded DNA ds (dA_20_T_20_) a strong negative CD band occurs because the chirality of the double-stranded DNA helix is transferred to fullerene **1** by electrostatic interactions. In comparison to **1**, fullerene **2** shows stronger CD due to the presence of the two 2′-deoxyuridine moieties that may form a chiral assembly in aqueous solution without any DNA template. Only the presence of the complementary template dA_20_ strengthens the CD, whereas no significant change of CD in the presence of the wrong template T_20_ or the double-stranded DNA ds (dA_20_T_20_) was observed. T_20_ does not provide the right binding sites for fullerene **2**, and in ds (dA_20_T_20_) the binding sites at dA_20_ are occupied by the counter strand T_20_. Altogether, this shows that **2** binds only to the complementary dA_20_ and indicates the canonical base pairing of the 2′-deoxyuridines as part of fullerene **2** with the 2′-deoxyadenosines as part of the template dA_20_. This makes fullerene **2** a potentially better component for supramolecular DNA architectures compared to fullerene **1** that binds non-specifically and simply by electrostatic interactions, as the specific interactions could be used to define charge carrier percolation pathways to the respective electrodes in a solar cell.

### Fluorescence Quenching of DNA-Based Chromophore Assemblies

**FIGURE 4:**
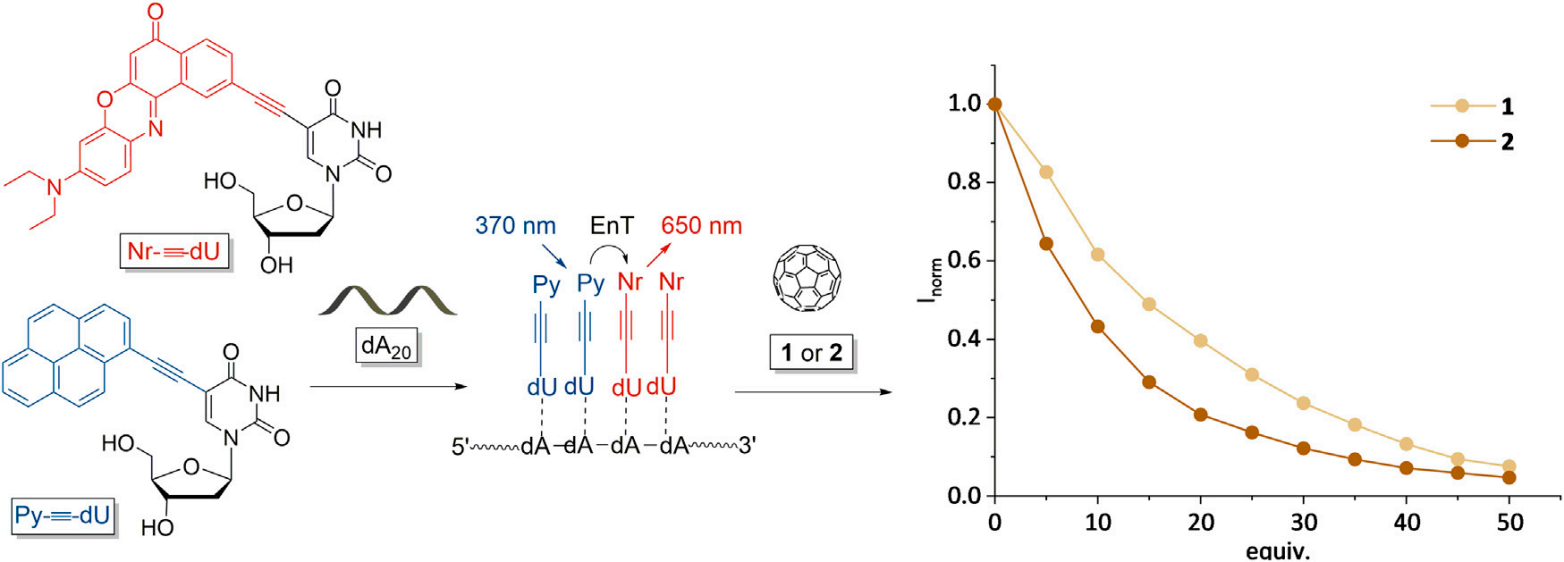
Fluorescence (I) quenching of the supramolecular DNA assemblies consisting of 8 Py-≡-dU:12 Nr-≡-dU units and dA_20_ as DNA template by different amounts (equiv. with respect to the amount of DNA) of fullerenes **1** and **2**. Excitation of Py-≡-dU at 380 nm induces an energy transfer (EnT) to Nr-≡-dU that emits at 650 nm.

An important prerequisite of the DNA-based chromophore architectures as light-harvesting systems for optoelectronic devices is their dual color fluorescence that is induced by energy transfer processes between the chromophores. When brought in contact with fullerenes, the fluorescence is quenched, due to exciton splitting on the chromophore and electron transfer to the fullerenes. In order to compare the fluorescence quenching properties of fullerenes **1** and **2**, we applied them to a DNA-templated chromophore assembly comprising Py-≡-dU and Nr-≡-dU as chromophore-2′-deoxyuridine conjugates that assemble along dA_20_ as single-stranded DNA templates (Figure 4). We published this type of chromophore architecture previously (Ensslen et al., 2015). Both chromophore conjugates are 2′-deoxyuridines and thus bind statistically to the 2′-deoxyadenosines in the DNA template, i.e., the chromophore sequence cannot be controlled. According to our published results this ratio showed the most efficient energy transfer between both chromophore-nucleoside conjugates (Ensslen et al., 2016). The DNA template A_20_ provides the ideal length for preparation of the mixed chromophore assembly (Ensslen et al., 2015). Shorter templates (like dA_10_) do not efficiently form chromophore assemblies, longer templates are difficult to handle due to solubility issues. In analogy to our previous solar cell experiments (Ensslen et al., 2016), we tested the fluorescence quenching with a DNA-templated assembly of a ratio of 8 Py-≡-dU:12 Nr-≡-dU units and dA_20_. The DNA assemblies were prepared by stock solutions of the chromophore-2′-deoxynucleotides in DMSO that were added to the DNA templates in aqueous solution. The excess of chromophore-2′-deoxynucleoside conjugates that were not bound to the DNA template was not soluble in aqueous solution and was removed by centrifugation (according to our published procedures) (Ensslen et al., 2015). Finally, the fullerenes **1** and **2** were prepared in separate stock solutions in DMSO and titrated as 5 equivalent aliquots (based on the number of binding sites for the chromophore nucleosides in the template) to the DNA assemblies. In both cases, fluorescence quenching was observed (Figure 4), but there are significant differences. The fluorescence quenching by fullerene **2** is stronger than by fullerene **1** which can be attributed to the more specific binding of fullerene **2** to the DNA-based chromophore assembly, which could lead to a closer proximity of fullerene **2** and the chromophores. As fullerene **1** binds electrostatically to the backbone of dA_20_ at larger distance, charge carrier transfer from the chromophores to the fullerenes might be reduced. Unbound fullerenes could not be removed by centrifugation. We note that chromophore quenching by fullerenes is unlikely to occur if the fullerenes are not bound to the DNA template, due to their low concentration in the aqueous solution and the low probability for collisional quenching.

### Optical Characterization of Thin Films

**FIGURE 5:**
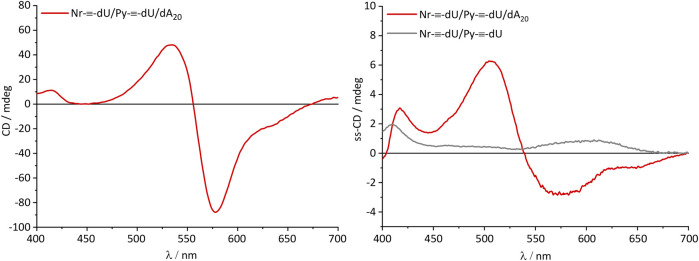
CD of the chromophore-DNA aggregates in solution (**left**, 1.5 µm DNA) and solid state-CD of the chromophore aggregates in thin films w/and w/o DNA template **(right)**. Below 400 nm the CD signal was not discernible due to the strong absorption of the chromophores in this regime, leading to a very weak signal at the detector.

Most critical for optoelectronic applications is the question whether or not the supramolecular structure of the DNA-based assemblies is maintained when the solid phase is prepared from solution. Commonly, this case is assumed, and consequently supramolecular assemblies are only characterized in solution. Notably, optical spectroscopy of solid materials can be experimentally challenging. We characterized the CD of thin films that were prepared from dA_20_-DNA assemblies comprising 8 Py-≡-dU:12 Nr-≡-dU. For the thin films, the chromophore nucleosides and DNA were dissolved in DMSO/water mixtures 4/1 (v/v) in a concentration of 40 mg/mL. Quartz glass plates were coated with 50 µL of this solution by drop casting and dried at 60°C in vacuum. For reference, a second set of thin films was prepared from the chromophore conjugate mixtures, but without any DNA template. The CD spectra were recorded in 45° rotations of the quartz glass plate (Supplementary Figure S3). A total of seven different angles were measured in order to minimize spectral artifacts. The spectra were averaged to eliminate influences from linear dichroism or scattering from inhomogeneous surfaces. The CD of the assembly consisting of Py-≡-dU, Nr-≡-dU and dA_20_ in solution is dominated by the strong excitonic signal in the nile red absorption range with a positive peak at 535 nm, a negative peak at 578 nm and a zero transition at 556 nm (Figure 5). This indicates a coplanar arrangement of Nr-≡-dU along the DNA template forming a left-handed helix according to the excitonically coupled CD. The CD of Py-≡-dU in the pyrene absorption range is smaller and shows a characteristic maximum at 415 nm. Strikingly, a very similar CD spectrum is observed for this type of DNA-based assembly also in the thin film. In particular, the excitonically coupled CD of the Nr-≡-dU components is obtained. In absence of the DNA template in the thin film, the CD of both chromophores shows different signals, in particular the strong exciton signal for Nr-≡-dU is not observed. This is an important evidence that a decent amount of chromophores remains bound to the DNA template, and hence that the DNA template provides structure even in the solid state. In particular the DNA-induced helicity in the chromophore assemblies is not only observed in solution, but also preserved in the solid phase.

### Light-Harvesting in DNA-Fullerene Assemblies

**FIGURE 6:**
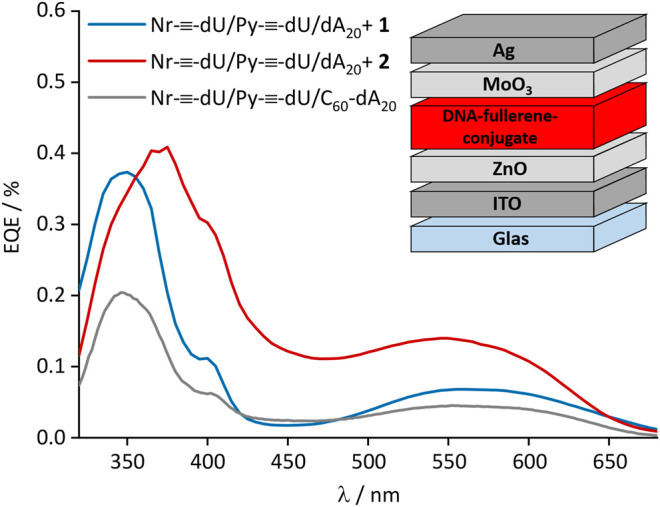
External quantum efficiency (EQE) of the DNA-based solar cells. Inset: Device architecture.

The strong fluorescence quenching of the DNA-based chromophore assemblies by the fullerenes **1** and **2** motivated their incorporation into organic solar cells. For best processibility, we opted for the inverted device architecture that is depicted in the inset of Figure 6. We chose an inverted device architecture as regular architectures commonly comprise PEDOT:PSS bottom transport layers, which is not water resistant, and the light-harvesting layer was deposited from a water/DMSO solution. Electrons were collected at the transparent indium tin oxide (ITO) electrode. Their extraction was facilitated by a 30 nm zinc oxide (ZnO) thin-film, spin cast from nanoparticle dispersion. Holes were extracted into the silver (Ag) anode, and the selectivity of the electrode was enhanced by introducing a vacuum deposited molybdenum oxide (MoO_3_) layer with a thickness of 10 nm. Detailed device fabrication instructions are provided in the Supplementary Information. The light-harvesting layer comprised the DNA template dA_20_ with a mixture of the chromophores Py-≡-dU:Nr-≡-dU (8:12) and the fullerenes **1** or **2** (20 equiv.) attached. The total number of chromophores deliberately exceeded the number of bases by a factor of two, likely resulting in some non-bound chromophore excess. Binding of all chromophores to the DNA would therefore only be possible if two chromophores bind to one adenosine as depicted in Figure 1. Together, all ingredients exhibited a broad spectral absorption of up to 700 nm. The conversion of photo-generated excitons into free charge carriers within the device was quantified by measurement of the external quantum efficiency (EQE, Figure 6), that is the number of charge carriers extracted at the electrodes divided by the number of incident photons. The broad range of the EQE spectra confirms that both chromophores Py-≡-dU and Nr-≡-dU as well as the fullerenes **1** and **2** contribute to the photocurrent generation. The peak in the EQE spectrum below 400 nm can be attributed to the absorption of the fullerenes and is of similar magnitude for both fullerene derivatives under investigation, fullerene **1** and **2**. However in the absorption regime of Py-≡-dU and Nr-≡-dU, the photocurrent is higher if fullerene **2** is employed. This relative enhancement indicates that fullerene **2** facilitates the dissociation of excitons on both Py-≡-dU and Nr-≡-dU. This observation is consistent with the fluorescence quenching efficiency in solution and can again be explained by a closer proximity of fullerene **2** to the chromophores for better charge carrier transition from the chromophores to the fullerenes. Although the fluorescence quenching was very strong, the EQE is limited to a maximum of 0.4%, most likely due to missing percolation pathways for the charge carriers toward the respective electrodes. As Py-≡-dU, Nr-≡-dU and 2 are statistically distributed along dA_20_, holes can be trapped on Py-≡-dU and Nr-≡-dU without a percolation pathways to the anode. Trapping of holes can lead to an enhanced recombination with electrons and hence a reduction of the extracted current. In the future, the extraction of holes might be enhanced by the sequence-selective assembly of the chromophores along the DNA template, ordered by electron acceptor or donator to facilitate undisturbed charge carrier transport to the respective electrodes. Likewise, electrons can be trapped on isolated fullerenes which do not provide a percolation pathway to the electrode. By comparing the EQE with the DNA assembly that consists of Py-≡-dU:Nr-≡-dU 8:12 and the fullerene-dA_20_ conjugate (Ensslen et al., 2016), we infer that increasing the fullerene content can enhance the EQE, presumably due to better electron percolation pathways. In this earlier DNA assembly, the fullerene content was low (chromophore:fullerene 20:1) because it was covalently attached to the DNA template. The non-covalently attached fullerene **2** therefore occurs closer to the chromophores and hence greatly enhances the photocurrent.

## Conclusion

We synthesized two fullerene derivates **1** and **2**. The literature-known fullerene **1** binds by electrostatic interactions to single-stranded DNA, and the new fullerene **2** contains two 2′-deoxyuridine moieties and assembles specifically along the dA_20_ DNA template. The DNA-directed assembly of both fullerenes in aqueous solution was investigated by UV/Vis absorbance and CD spectroscopy. The specific interactions with DNA make fullerene **2** a significantly better component for supramolecular DNA architectures than fullerene **1**. In order to compare the fluorescence quenching properties of both fullerenes, **1** and **2**, we applied either of them to a DNA-templated chromophore assembly consisting of Py-≡-dU and Nr-≡-dU as chromophore-2′-deoxynucleoside conjugates in a ratio of 8:12 that assemble along dA_20_ as single-stranded DNA templates. Fullerene **2** induces stronger fluorescence quenching. We characterized the CD of thin films that were prepared with the DNA assemblies consisting of 8 Py-≡-dU:12 Nr-≡-dU with dA_20_. The DNA template induces a helical chirality of the chromophore assemblies which transfers from solution to the solid phase. When implemented in DNA-based solar cells, fullerene **2** showed an enhanced external quantum efficiency compared to fullerene **1**, consistent with the stronger fluorescence quenching and, presumably, an enhanced exciton dissociation. To further enhance the photon-to-electron conversion efficiency, template specific binding could be used in the future to enhance the percolation pathways of the charge carriers toward the electrodes. This work clearly shows the potential of DNA as structural element for ordering chromophores into functional π-systems which may be employed in future organic optoelectronic devices.

## Additional Requirements

For additional requirements for specific article types and further information please refer to Author Guidelines.

## Data Availability

The original contributions presented in the study are included in the article/Supplementary Material, further inquiries can be directed to the corresponding author.
