# Supplementary material for: Molecular Chromophore-DNA Architectures With Fullerenes: Optical Properties and Solar Cells
Source: Front Chem. 2021 Feb 23;9:645006. doi: 10.3389/fchem.2021.645006 (PMC7941155; doi:10.3389/fchem.2021.645006)
Supplement: Supplementary file 1 [file presentation1.pdf]

Molecular Chromophore-DNA Architectures with Fullerenes: Optical Properties and Solar Cells

Sara Müller[1], Felix Manger[2,3], Lorenz Graf von Reventlow[2,3], Alexander Colsmann[2,3], Hans-Achim Wagenknecht[1*]

Supplementary Material

[1]Institute of Organic Chemistry, Karlsruhe Institute of Technology (KIT), Fritz-Haber-Weg 6, 76131 Karlsruhe, Germany

[2]Material Research Center for Energy Systems, Karlsruhe Institute of Technology (KIT), Strasse am Forum 7, 76131 Karlsruhe, Germany

[3]Lichttechnisches Institute, Karlsruhe Institute of Technology (KIT), Engesserstrasse 13, 76131 Karlsruhe, Germany

***Correspondence:**

Wagenknecht@kit.edu

1 Materials and Methods

Solvents & reagents

All used chemicals were of "for synthesis" purity. Used solvents for synthesis, optical spectroscopy or analysis were "HPLC" or "pro analysis". Deionized and ultrafiltrated water was obtained from a *Millipore Direct 8/16* from MERCK MILLIPORE. Unmodified DNA strands (HPLC-purified and lyophilized) were obtained from METABION. Concentrations of DNA solutions were determined with a NANODROP ND-100 spectrophotometer.

NMR spectroscopy

NMR spectra were recorded in deuterated solvents from EURISOTOP on a BRUKER *Advance 500* with tetramethylsilane (TMS, $\delta = 0.00$ ppm) as standard. Shifts are reported in parts per million (ppm) relative to the residual proton solvent peaks.

Optical spectroscopy

Spectra were recorded at 20 °C in semi-micro quartz glass cuvettes from STARNA (width 10 mm, volume 1.4 mL). The following instruments were used:

- Absorption: *Cary 100 Bio* from VARIAN
- Fluorescence: *Fluoromax-3* from JOBIN-YVON (λ_{exc} :380 nm, slits: 2 nm)
- Circular dichroism: JASCO *J-810 Spectropolarimeter* (scanning speed:100 nm/min, accumulations: 4, D.I.T.:4 s, band width: 4 nm)
- ss-CD: JASCO *J-810 Spectropolarimeter* (45° rotation, scanning speed:50 nm/min, accumulations: 1, D.I.T.:4 s, band width: 8 nm)

2 Synthesis and characterization

The compound **1**(Cassell et al., 1998), **3**(Abellán Flos et al., 2016), **4**(Hornum et al., 2015), Py-≡-dU(Astakhova et al., 2007) and Nr-≡-dU(Varghese et al., 2009) were synthesized according to literature.

Compound **5**

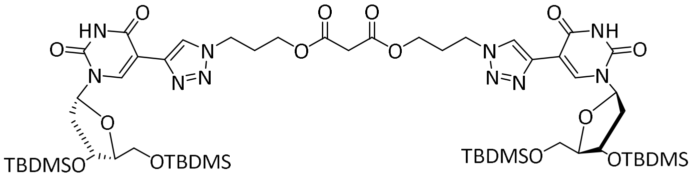

5

$C_{55}H_{94}N_{10}O_{14}Si_4$

1230.6028 g/mol

391 mg **3** (0.810 mmol, 2.20 equiv.) and 118 mg Tris((1-benzyl-4-triazolyl) methyl)amine (TBTA, 0.22 mmol, 0.600 equiv.) were dissolved. Successively, 41.4 mg Tetrakis(acetonitrile)copper(I) hexafluorophosphate (1.11 mmol, 0.300 equiv.) and 7.66 mg sodium ascorbate (1.11 mmol, 0.300 equiv.) were added. Subsequently, 100 mg **4** (0.372 mmol, 1.00 equiv.), dissolved in DCM, were added dropwise and the resulting solution was stirred at room temperature for 16 hours. After dilution with DCM, the solution was washed three times with water. The organic phase was dried over sodium sulfate, concentrated under reduced pressure and purified via column chromatography (hexane /ethyl acetate 1:1 → 0:1). The product was obtained as colorless foam (419 mg, 0.34 mmol, 92 %).

DC (EE): R_f = 0.75.

^{1}H NMR (500 MHz, $CDCl_3$) δ = 9.54 (s, 2H, N*H*), 8.52 (s, 2H, CH_{arom}), 8.32 (s, 2H, CH_{arom}), 6.34 (dd, J=7.9 Hz, 5.8 Hz, 2H, 1´-C*H*), 4.51 (t, J=6.8 Hz, 4H, CH_2), 4.45 (dt, J=4.9 Hz, 2.2 Hz, 2H, 3´-C*H*), 4.19 (t, J=5.9 Hz, 4H, CH_2), 4.01 (q, J=3.7 Hz, 2H, 4´-C*H*), 3.87 – 3.75 (m, 4H, 5´-CH_2), 3.47 (s, 2H, CH_2), 2.37 (dd, J=5.8 Hz, 2.3 Hz, 2H, 2´-CH_2), 2.36 – 2.28 (m, 4H, CH_2), 2.15 (ddd, J=13.5 Hz, 8.0 Hz, 5.9 Hz, 2H, 2´-CH_2), 0.90 (s, 18H, CH_3), 0.85 (s, 18H, CH_3), 0.10 (d, J=1.9 Hz, 12H, CH_3), 0.09 (s, 6H, CH_3), 0.08 (s, 6H, CH_3).

^{13}C NMR (126 MHz, $CDCl_3$) δ = 166.5 (C_q), 161.7 (C_q), 149.7 (C_q), 139.2 (C_q), 136.5 (4CH_{arom}), 123.0 (CH_{arom}), 106.1 (C_q), 88.5 (4'CH), 86.2 (1'CH), 72.9 (3'CH), 63.4 (5'CH), 62.2 (CH_2), 46.9 (CH_2), 41.3 (2'-CH_2), 41.3 (CH_2), 29.3 (CH_2), 26.1 (CH_3), 25.9 (CH_3), 18.6 (C_q), 18.2 (C_q), -4.5 (CH_3), -4.7 (CH_3), -5.2 (CH_3), -5.4 (CH_3).

HRMS-ESI (m/z): Calcd. for $C_{55}H_{95}N_{10}O_{14}Si_4$ [MH^+]: 1231.6101; found: 1231.6100.

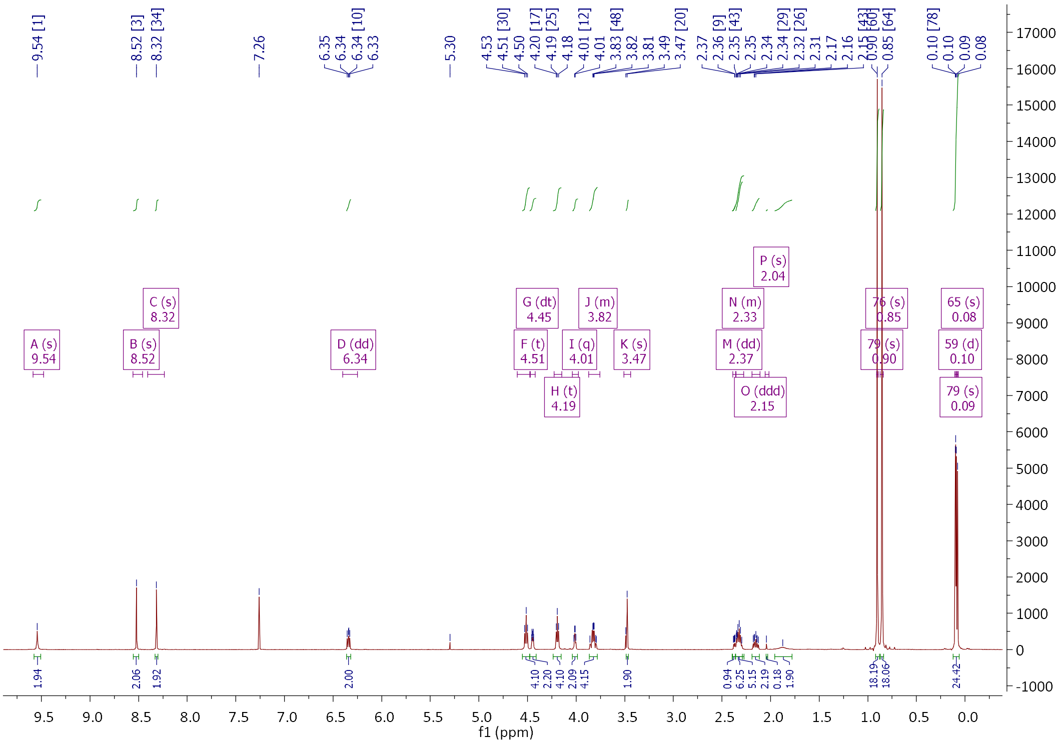
A (s) 9.54
B (s) 8.52
C (s) 8.32
D (dd) 6.34
F (t) 4.51
G (dt) 4.45
H (t) 4.19
I (q) 4.01
J (m) 3.82
K (s) 3.47
M (dd) 2.37
N (m) 2.33
O (ddd) 2.15
P (s) 2.04
76 (s) 0.85
79 (s) 0.90
65 (s) 0.08
59 (d) 0.10
79 (s) 0.09
f1 (ppm)

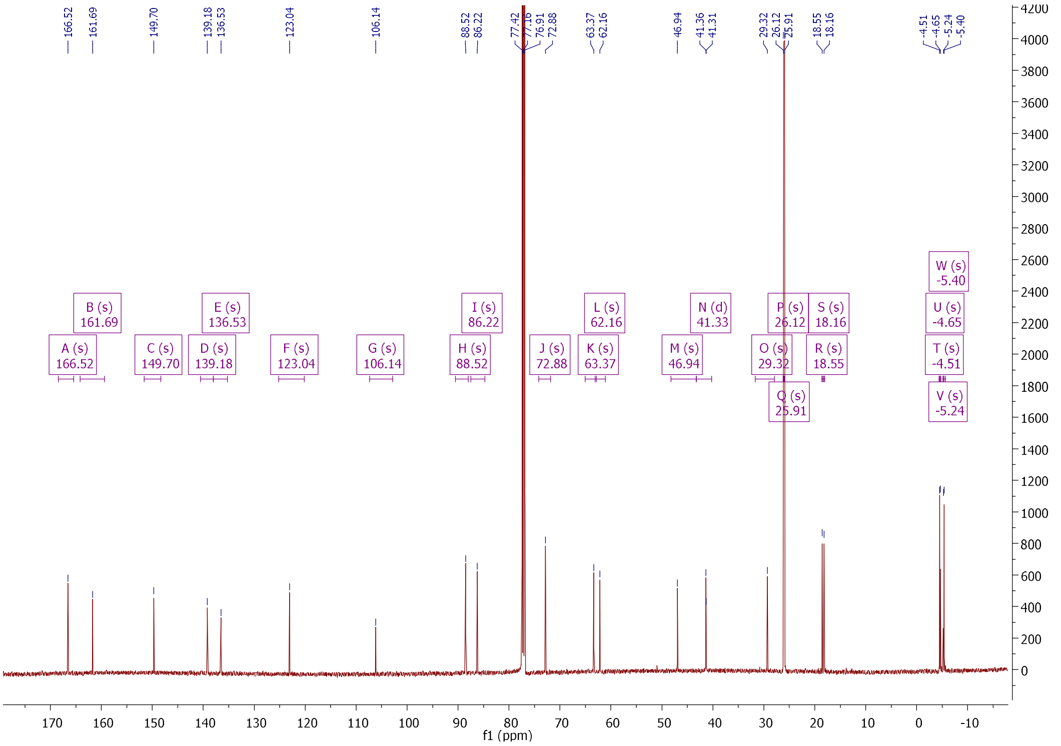
A (s) 166.52
B (s) 161.69
C (s) 149.70
D (s) 139.18
E (s) 136.53
F (s) 123.04
G (s) 106.14
H (s) 88.52
I (s) 86.22
J (s) 72.88
K (s) 63.37
L (s) 62.16
M (s) 46.94
N (d) 41.33
O (s) 29.32
P (s) 26.12
Q (s) 25.91
R (s) 18.55
S (s) 18.16
T (s) -4.51
U (s) -4.65
V (s) -5.24
W (s) -5.40
f1 (ppm)

Compound **6**

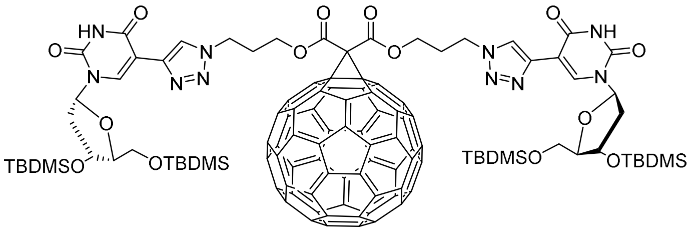

6

$C_{115}H_{92}N_{10}O_{14}Si_4$

1950.6028 g/mol

200 mg **5** (0.16 mmol, 1.00 equiv.), 117 mg C_{60}-fullerene (0.16 mmol, 1.00 equiv.) and 82.5 mg iodine (0.330 mmol, equiv.) were dissolved in toluene. After complete dissolution, 49.1 µL 1,8-diazabicyclo[5.4.0]undec-7-ene (DBU, 50.1 mg, 0.33 mmol, 2.00 equiv.) were added and the solution was stirred at room temperature for 16 h. The reaction mixture was concentrated under reduced pressure and purified via column chromatography (DCM/MeOH = 1/0 → 40/1). The product was obtained as brown solid (106 mg 0.05 mmol, 34 %).

DC (EE/Hex = 3:1): R_f = 0.51.

^{1}H NMR (500 MHz, $CDCl_3$): δ (ppm) = 9.54 (s, 2H, N*H*), 8.52 (s, 2H, CH_{arom}), 8.42 (s, 2H, CH_{arom}), 6.38 – 6.25 (m, 2H, 1´-C*H*), 4.61 (dt, J = 12.2 Hz, J = 6.3 Hz, 8H, CH_2), 4.45 (m, 2H, 3´-C*H*), 4.01 (d, J = 2.3 Hz, 2H, 4´-C*H*), 3.82 (d, J = 3.2 Hz, 4H, 5´-CH_2), 2.54 (p, J = 6.5 Hz, 4H, CH_2), 2.35 (ddd, J = 12.8 Hz, 5.4 Hz, 1.8 Hz, 2H, 2´-CH_2), 2.18 (m, 2H, 2´-CH_2), 0.90 (s,18 H, CH_3), 0.86 (s,18 H, CH_3), 0.09 (dd, J = 3.5, 2.3 Hz, 24 H, CH_3).

^{13}C NMR (126 MHz, $CDCl_3$): δ (ppm) = 163.5 (C_q), 161.6 (C_q), 149.6 (C_q), 145.5 (C_{60}), 145.4 (C_{60}), 145.2 (C_{60}), 145.1 (C_{60}), 145.0 (C_{60}) 144.8 (C_{60}), 144.7 (C_{60}), 144.0 (C_{60}), 143.2 (C_{60}), 143.1 (C_{60}), 143.1 (C_{60}), 142.3 (C_{60}), 141.9 (C_{60}), 141.1 (C_{60}), 139.5 (C_{60}), 139.1 (C_q), 136.3 (4CH_{arom}), 122.8 (CH_{arom})*,* 106.2 (C_q), 88.5 (4‘CH), 86.3 (1‘-CH), 72.9 (3‘-CH), 71.3 (5‘-CH), 64.1 (CH_2), 63.4 (CH_2), 51.7 (C_q), 47.0(CH_2), 41.3 (2’-CH_2), 29.5 (CH_2), 26.2 (CH_3), 25.9 (CH_3), 18.6 (C_q), 18.0 (C_q), -4.5 (CH_3), -4.6 (CH_3), -5.2 (CH_3), -5.6 (CH_3).

HRMS-ESI (m/z): Calcd. for $C_{115}H_{92}N_{10}O_{14}Si_4$ [M^+]: 1950.6028; found: 1950.5967

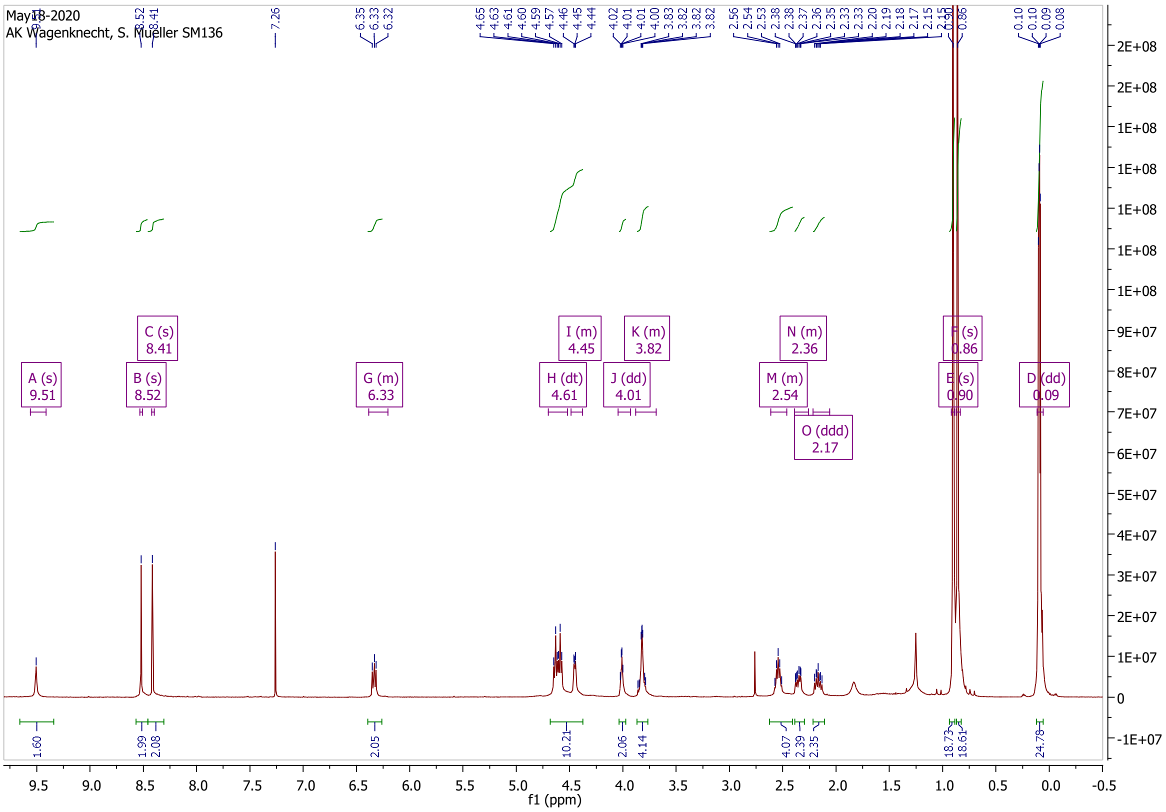
May18-2020
AK Wagenknecht, S. Mueller SM136
f1 (ppm)

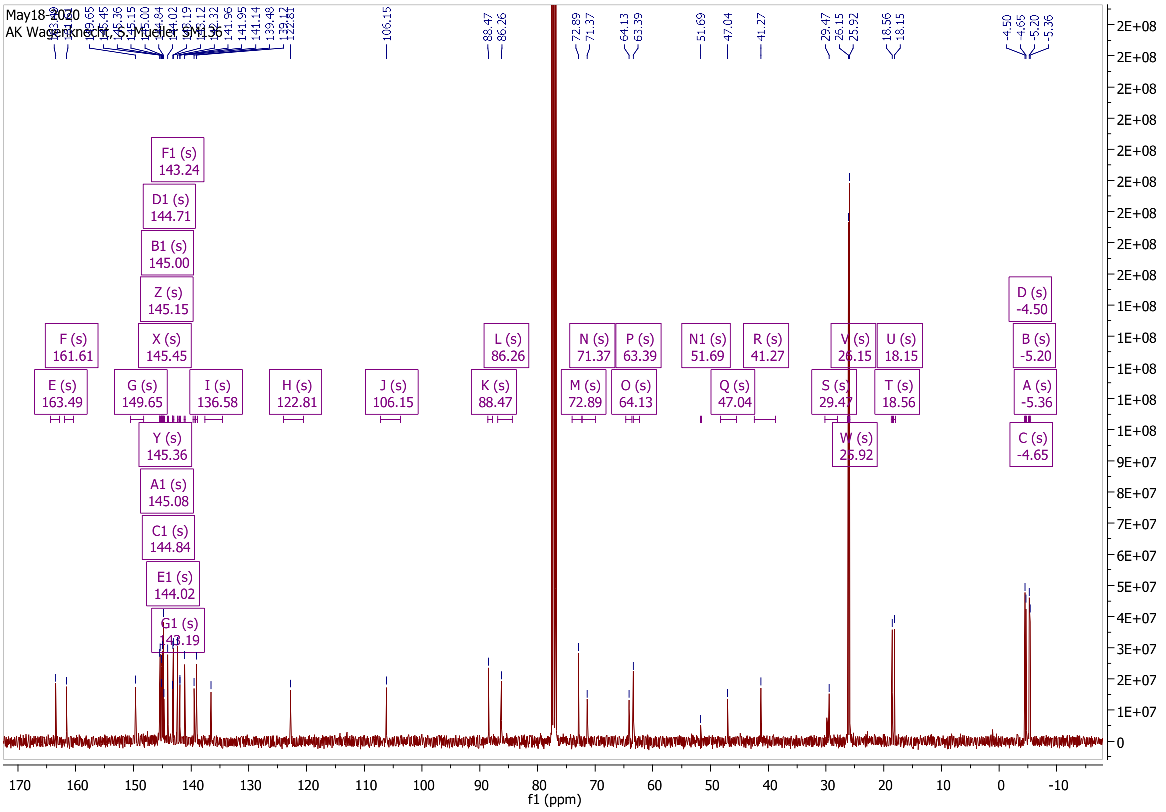
May18-2020
AK Wagenknecht, S. Mueller SM136
f1 (ppm)

Compound **2**

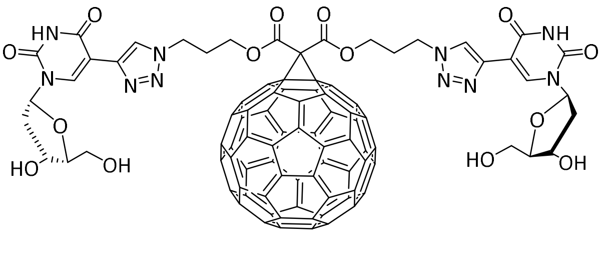

2

$C_{91}H_{36}N_{10}O_{14}$

1494.2569 g/mol

In a tightly closed crimp vial 226 mg **10** (0.116 mmol, 1.00 equiv.) were dissolved in dimethylformamide. Subsequently, 190 µL $Et_3N{\cdot}3HF$ (187 mg, 1.16 mmol, 10.0 equiv.) were added and the reaction mixture were stirred at room temperature for 72 hours. The solvent was removed under reduced pressure and the residue was dispersed in cold methanol and centrifuged. The supernatant was discarded and the obtained solids were dried in high vacuum. The product was obtained as brown solid (130 mg, 0.087 mmol, 75 %).

DC (DCM/MeOH 10:1): $R_f = 0.44$.

^{1}H NMR (500 MHz, DMSO-d6): δ (ppm) = 11.63 (s, 2H, N*H*), 8.51 (s, 2H, CH_{arom}), 8.45 (s, 2H, CH_{arom}), 6.22 (t, *J* = 6.8 Hz, 2H, 1´-C*H*), 5.28 (d, *J* = 4.1 Hz, 2H, 3´-OH), 5.00 (t, *J* = 4.9 Hz, 2H, 5´-OH) 4.63 (t, *J* = 6.5 Hz, 4H, CH_2), 4.55 (m, 4H, CH_2), 4.27 (s, 2H, 3´-C*H*), 3.84 (m, 2H, 4´-CH_2), 3.59 (m, 4H, 5´-CH_2), 2.40 (t, *J*=21.2 Hz, 4H, CH_2), 2.17 (s, 4H, 2´-CH_2).

^{13}C-NMR (126 MHz, DMSO-*d*6): δ (ppm) = 162.5 (C_q), 162.2 (C_q), 149.6 (C_q), 145.0 (C_{60}), 144.7 (C_{60}), 144.6 (C_{60}), 144.3 (C_{60}), 144.2 (C_{60}), 144.1 (C_{60}), 144.0 (C_{60}), 143.3 (C_{60}), 142.5 (C_{60}), 142.4 (C_{60}), 141.6 (C_{60}), 141.6, 141.3 (C_{60}), 141.2 (C_{60}), 140.3 (C_{60}), 138.9 (C_q), 138.4 (C_{60}), 138.4 (C_{60}), 136.0 (4CH_{arom}), 128.3 (C_{60}), 122.6 (CH_{arom}), 105.4 (C_q), 87.6 (4‘-CH), 84.7 (1‘-CH), 70.8 (3‘-CH),71.1 (5‘-CH_2), 64.9 (CH_2), 61.5 (CH_2), 51.7 (C_q), 46.8 (CH_2), 28.8 (CH_2).

HRMS-ESI (m/z): Calcd. for $C_{91}H_{38}N_{10}O_{14}$ [M^+]: 1948.2569; found: 1494.2526.

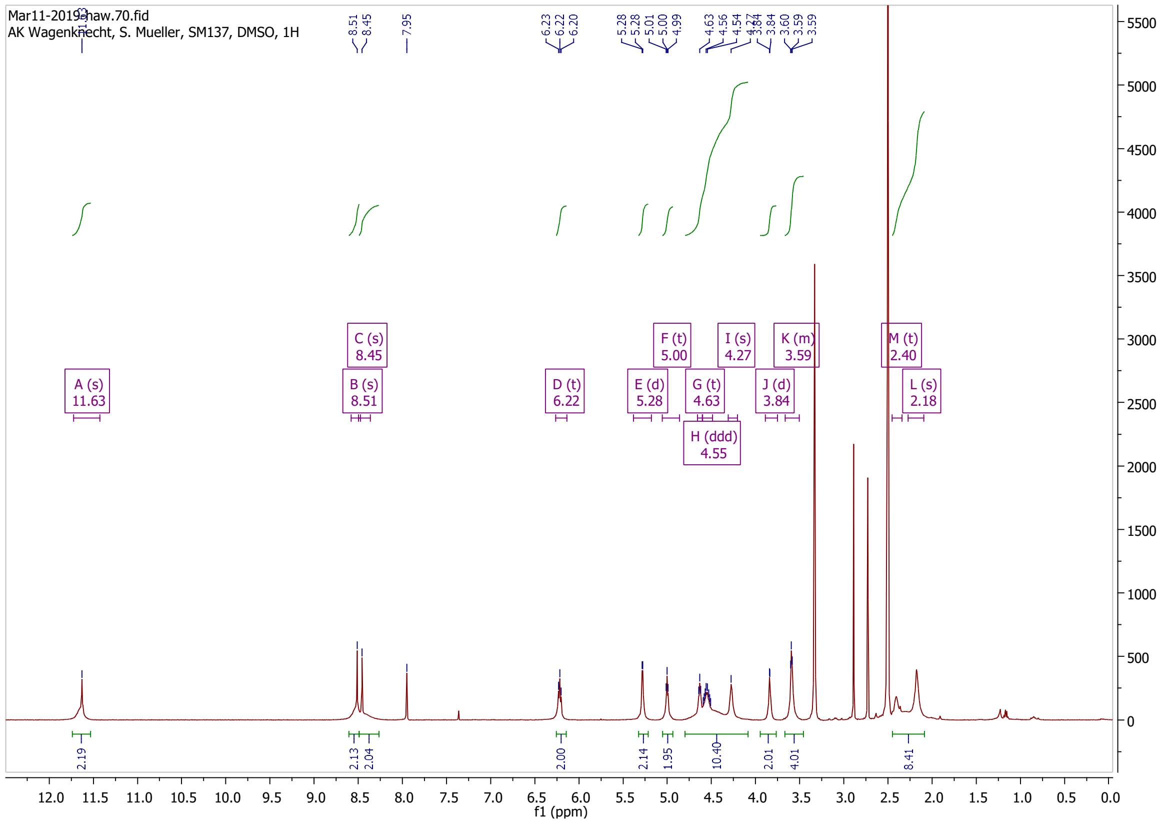
Mar11-2019-haw.70.fid
AK Wagenknecht, S. Mueller, SM137, DMSO, 1H
A (s) 11.63
B (s) 8.51
C (s) 8.45
D (t) 6.22
E (d) 5.28
F (t) 5.00
G (t) 4.63
H (ddd) 4.55
I (s) 4.27
J (d) 3.84
K (m) 3.59
L (s) 2.18
M (t) 2.40
f1 (ppm)

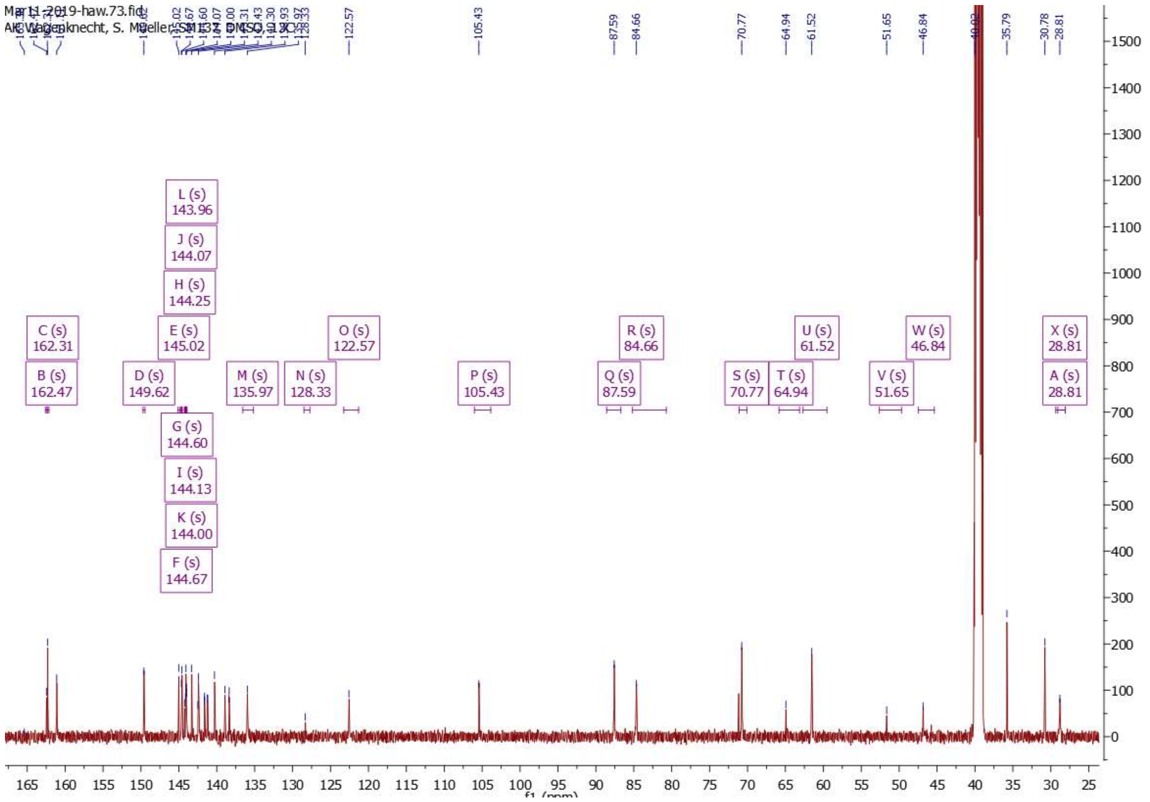
Mar11-2019-haw.73.fid
AK Wagenknecht, S. Mueller, SM137, DMSO, 13C
A (s) 28.81
B (s) 162.47
C (s) 162.31
D (s) 149.62
E (s) 145.02
F (s) 144.67
G (s) 144.60
H (s) 144.25
I (s) 144.13
J (s) 144.07
K (s) 144.00
L (s) 143.96
M (s) 135.97
N (s) 128.33
O (s) 122.57
P (s) 105.43
Q (s) 87.59
R (s) 84.66
S (s) 70.77
T (s) 64.94
U (s) 61.52
V (s) 51.65
W (s) 46.84
X (s) 28.81

3 Sample Preparation

For all measurements in solution, ssDNA and dsDNA were used in a concentration of 0.75 µM. The fullerene derivatives were added stoichiometrically from a 2 mM stock solution in DMSO to the number of binding sites in the template. This resulted in fullerene concentration of 15 µM for all 20mer templates. The samples were incubated at 20 °C and then examined spectroscopically.

For the titration experiments, the DNA-dye aggregates (1.5 µM DNA) were prepared in water with 2% DMSO, according to the appropriate assembly protocol (Ensslen et al., 2015). Subsequently, the fullerene derivatives **1** and **2** were added directly from the respective 2 mM stock solution.

4 Additional Spectra

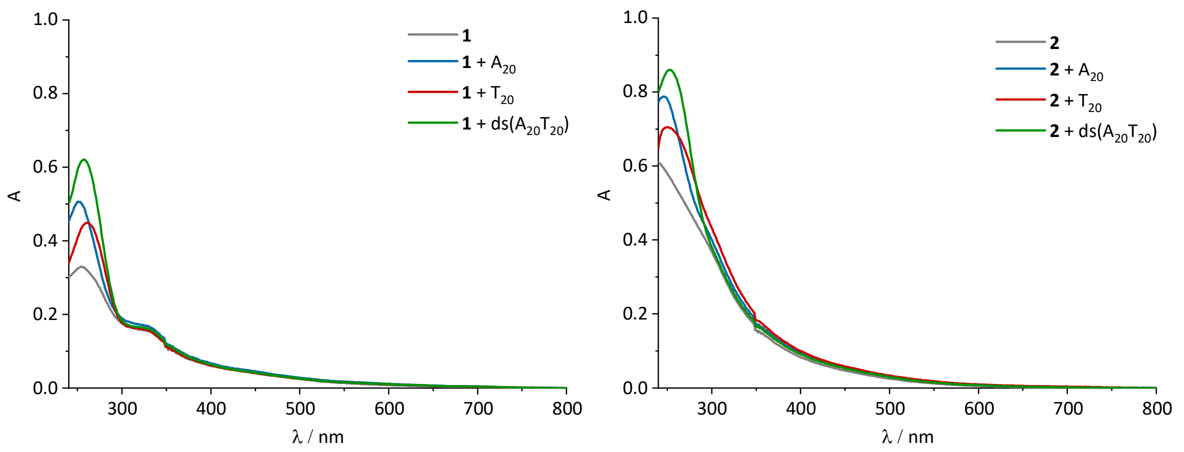

Supplementary Figure 1: UV/VIS spectra of 1 (left) and 2 (right), each 15.0 µM in aqueous solution (2% DMSO), and in the presence of 0.75 µM A_{20}, 0.75 µM T_{20} or 0.75 µM ds($A_{20}T_{20}$).

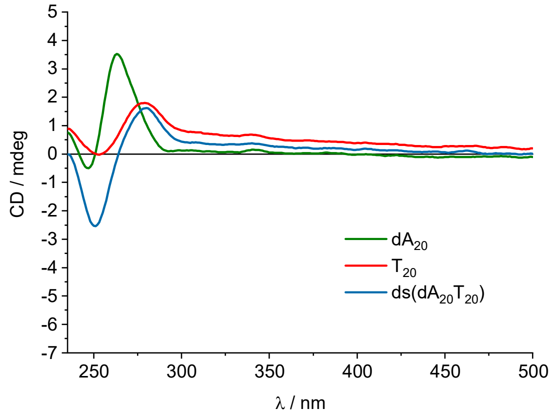

Supplementary Figure 2: CD spectra of 0.75 µM A_{20}, 0.75 µM T_{20} or 0.75 µM ds($A_{20}T_{20}$) in aqueous solution (2% DMSO).

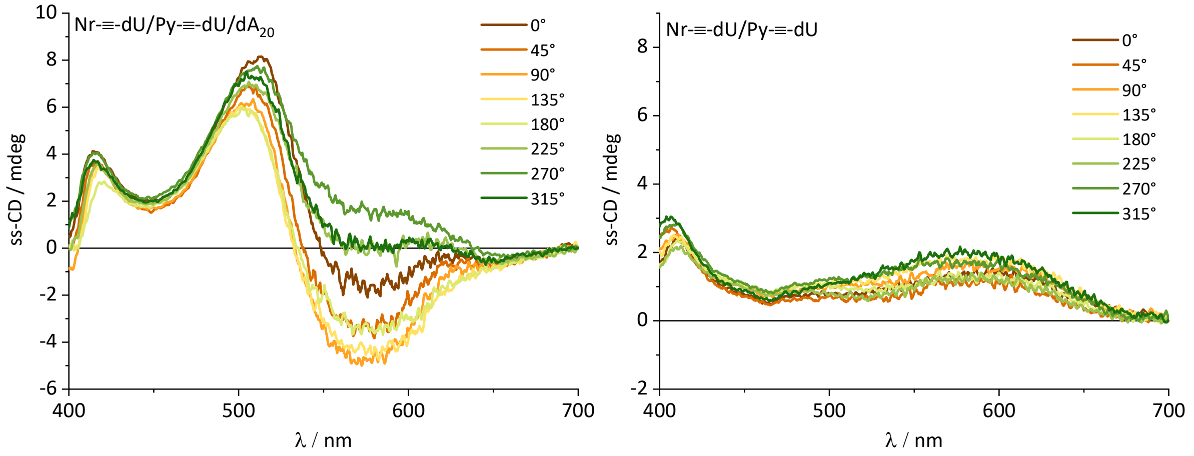

Supplementary Figure 3: Solid state-CD (ss-CD) of the chromophore aggregates in thin films w/ (left) and w/o DNA template (right). Below 400 nm the CD signal was not discernible due to the strong absorption of the chromophores in this regime, leading to a very weak signal at the detector

5 Preparation and characterization of the solar cells

Solar cells were fabricated using an inverted device architecture (ITO / ZnO / fullerene-chromophore-DNA mixture / MoO_x / Ag). Patterned indium tin oxide (ITO, 125 nm) coated glass substrates were cleaned by consecutive ultra-sonication in acetone and isopropanol (10 min) and exposition to an oxygen plasma (2 min). Zinc oxide nanoparticles (ZnO, formulation N-10, Avantama, diluted with isopropanol to 1wt%, 30 nm) were doctor bladed using an Universal Applicator (ZUA 2000, Zehntner

Testing Instruments, velocity 18 mm/s, gap height 50 µm, platen temperature 35°C, volume 30 µl) and annealed on a hotplate (10 min, 80°C). The fullerene-chromophore-DNA mixture was coated using a cylindrical applicator (diameter 10 mm, velocity 10 mm/s, gap height 150 µm, platen temperature 65 °C, volume 50 µl). The MoO_x (10 nm)/silver (100 nm) electrode was evaporated in high vacuum (10^{-6} mbar). The cross section of the ITO and the silver electrodes determine a photo-active area of 0.105 cm^2.

The photovoltaic performance of the solar cells was characterized with a home-built external quantum efficiency setup. Monochromatic light was generated by a Czerny-turner-monochromator (Omni-λ300, LOT Oriel with the MSZ3122, LOT Oriel filter wheel), that sequentially filtered the light of a xenon high-pressure lamp (450W LSH601. LOT Oriel). The light was optically chopped (C-995, Terahertz Technologies) with 373 Hz to allow for a lock-in amplification. The output light was split into two beams. One beam was monitored by a monitor diode (K1713-09, Hamamatsu) to track fluctuations of the xenon high pressure lamp. The other beam was coupled into an optical fiber (fiber patch cable M37L02, Thorlabs) and focused onto the measurement sample inside of a nitrogen glovebox to measure in inert conditions. The photocurrents of the monitor diode and the sample were amplified by two transimpedance amplifiers (OE-200S, Femto Messtechnik) with an amplification of 10^5 and 10^8, respectively. The amplified signal was measured by a lock-in amplifier (eLockIn 203 von Anfatec Instruments) with a settling time of two seconds and two seconds integration time. The setup was referenced to a photodiode (SM1PD2A-CAL, Thorlabs, certified by PTB 2145 PTB 08) to calculate the spectral response.

6 References

Abellán Flos, M., García Moreno, M.I., Ortiz Mellet, C., García Fernández, J.M., Nierengarten, J.-F., and Vincent, S.P. (2016). Potent Glycosidase Inhibition with Heterovalent Fullerenes: Unveiling the Binding Modes Triggering Multivalent Inhibition. *Chemistry – A European Journal* 22**,** 11450-11460.

Astakhova, I.V., Malakhov, A.D., Stepanova, I.A., Ustinov, A.V., Bondarev, S.L., Paramonov, A.S., and Korshun, V.A. (2007). 1-Phenylethynylpyrene (1-PEPy) as Refined Excimer Forming Alternative to Pyrene: Case of DNA Major Groove Excimer. *Bioconjugate Chemistry* 18**,** 1972-1980.

Cassell, A.M., Scrivens, W.A., and Tour, J.M. (1998). Assembly of DNA/Fullerene Hybrid Materials. *Angewandte Chemie International Edition* 37**,** 1528-1531.

Ensslen, P., Fritz, Y., and Wagenknecht, H.-A. (2015). Mixed non-covalent assemblies of ethynyl nile red and ethynyl pyrene along oligonucleotide templates. *Organic & Biomolecular Chemistry* 13, 487-492.

Hornum, M., Kumar, P., Podsiadly, P., and Nielsen, P. (2015). Increasing the Stability of DNA:RNA Duplexes by Introducing Stacking Phenyl-Substituted Pyrazole, Furan, and Triazole Moieties in the Major Groove. *The Journal of Organic Chemistry* 80, 9592-9602.

Varghese, R., Gajula, P.K., Chakraborty, T.K., and Wagenknecht, H.-A. (2009). Thieme Chemistry Journal Awardees - Where Are They Now?Synthesis and Optical Properties of Nile Red Modified 2′-Deoxyuridine and 7-Deaza-2′-deoxyadenosine: Highly Emissive Solvatochromic Nucleosides. *Synlett* 2009, 3252-3257.
